# The plasticizer butyl benzyl phthalate induces genomic changes in rat mammary gland after neonatal/prepubertal exposure

**DOI:** 10.1186/1471-2164-8-453

**Published:** 2007-12-06

**Authors:** Raquel Moral, Richard Wang, Irma H Russo, Daniel A Mailo, Coral A Lamartiniere, Jose Russo

**Affiliations:** 1 Breast Cancer Research Laboratory, Fox Chase Cancer Center, Philadelphia, PA 19111, USA; 2 Department of Pharmacology and Toxicology, University of Alabama at Birmingham, Birmingham, AL 35294, USA

## Abstract

**Background:**

Phthalate esters like n-butyl benzyl phthalate (BBP) are widely used plasticizers. BBP has shown endocrine-disrupting properties, thus having a potential effect on hormone-sensitive tissues. The aim of this study is to determine the effect of neonatal/prepubertal exposure (post-natal days 2–20) to BBP on maturation parameters and on the morphology, proliferative index and genomic signature of the rat mammary gland at different ages of development (21, 35, 50 and 100 days).

**Results:**

Here we show that exposure to BBP increased the uterine weight/body weight ratio at 21 days and decreased the body weight at time of vaginal opening. BBP did not induce significant changes on the morphology of the mammary gland, but increased proliferative index in terminal end buds at 35 days and in lobules 1 at several ages. Moreover, BBP had an effect on the genomic profile of the mammary gland mainly at the end of the exposure (21 days), becoming less prominent thereafter. By this age a significant number of genes related to proliferation and differentiation, communication and signal transduction were up-regulated in the glands of the exposed animals.

**Conclusion:**

These results suggest that BBP has an effect in the gene expression profile of the mammary gland.

## Background

Butyl benzyl phthalate (BBP) is a plasticizer commonly used in pipes, vinyl floor tiles, vinyl foams, and carpet backing, and to a minor extent, in cellulose plastics and polyurethane [1]. This compound has shown to be weakly estrogenic [2], and to induce adverse effects on the development of male reproductive system [3-5], thus acting as an endocrine disruptor. Adult BBP intake has been estimated at 2 μg/kg body weight/day and it has been stated that exposure to infants and children could be up to three-fold higher [1]. Environmental endocrine disruptors are of growing concern due to the potential impact on human health. Actually, BBP is one of the phthalates selected by the National Toxicology Program (NTP) Center for the Evaluation of Risks to Human Reproduction (CERHR), because of its high production volume, potential for human exposure from their widespread use and occurrence within the environment, concern to the public, and published evidence of reproductive or developmental toxicity [6]. The evaluation concluded that there was negligible concern for adverse reproductive effects in exposed men, but data was insufficient to reach conclusions on possible reproductive effects in exposed women [6]. Nevertheless, the European Commission has banned BBP from toys and child care items at concentrations of greater than 0,1% by mass of the plasticized material [7] and new data is providing demonstration of possible developmental effects in humans [8]. Urinary concentrations of phthalate metabolites in pregnant mothers, including the major BBP metabolite monobenzyl phthalate, have been inversely correlated to anogenital distance among male infants [8]. Moreover an etiological association of serum BBP concentration with endometriosis has been suggested, as severity of endometriosis was strongly correlated with BBP concentration in blood [9]. Although more data is needed regarding the possible effects of this compound on human health, experimental studies have given evidence of an antiandrogenic effect of BBP and monobenzyl phthalate. Prenatal and prepubertal exposure of rats to such compounds induced significant alterations on reproductive system of males, like undescended testes, decrease in the anogenital distance and other malformations of the external genitalia, sex accessory glands, epididymis and testes [3-5]. It has also been reported a decrease in the weight of the ovaries in females exposed during adulthood, as well as changes in the offspring of exposed rats, including a decrease of the body weight of male and female at birth and an increase in the anogenital distance in females [10]. All these changes are compatible with an endocrine disrupting action of BBP.

Endocrine disruptors have also been suggested to contribute to the development of hormone-dependent cancers [11]. Although the risk for cancer is multifactorial, there is substantial contribution of environmental factors, including diet and environmental chemicals. Breast cancer is an estrogen-dependent malignancy with a significant mortality in women worldwide [12]. Changes in the hormonal environment during critical stages of development can modify the architecture and biological characteristics of the mammary gland and thus affect the future susceptibility to develop breast cancer [13,14]. The effects of environmental endocrine disruptors seem to be dependent on the time when the exposure occurs. Diethylstilbestrol has been associated to increased or decreased susceptibility for chemically-induced mammary cancer in rats depending on the period of exposure [15,16].

The present study is designed to determine the effects of neonatal and prepubertal exposure to BBP on rat mammary gland at different stages of development using morphological parameters of gland differentiation [13,14], proliferative index and the genomic expression profile. Here we report that BBP has an effect on the gene expression profile of the mammary gland.

## Results

### Maturation parameters

Neonatal/prepubertal exposure to BBP did not affect body weight, uterine weight or day at vaginal opening. The uterine to body weight ratios were significantly increased in the exposed animals at 21 days of age (Table 1). BBP exposure did not modify the day at vaginal opening (control group: 30.46 ± 0.13 days; BBP group: 30.44 ± 0.11 days), but decreased significantly (p < 0.05) the body weight at that day (control group: 101.96 ± 1.09 g; BBP group: 97.26 ± 1.49 g).

**Table 1:** Parameters for post-natal maturation and development

	21 days	35 days	50 days	100 days
Uterine weight (mg)				
Control	30.0 ± 1.4	167 ± 27	288 ± 17	409 ± 20
BBP	36.0 ± 2.1	207 ± 16	319 ± 24	429 ± 19
Uterine weight/BW (mg/g)				
Control	0.51 ± 0.03	1.27 ± 0.20	1.48 ± 0.08	1.31 ± 0.07
BBP	0.65 ± 0.04*	1.56 ± 0.13	1.65 ± 0.10	1.39 ± 0.04
Development of the mammary gland				
Number of TEB				
Control	39.5 ± 2.32	30.4 ± 5.22	18.3 ± 3.71	0.22 ± 0.22
BBP	40.0 ± 4.68	30.2 ± 5.78	22.7 ± 3.16	0.40 ± 0.40
Number of TD				
Control	24.5 ± 1.65	38.2 ± 4.17	41.1 ± 3.40	57.3 ± 5.21
BBP	25.9 ± 2.52	33.5 ± 4.08	46.6 ± 7.31	61.8 ± 5.41
Number of AB				
Control	8.0 ± 1.87	14.9 ± 1.08	10.1 ± 2.04	0.2 ± 0.22
BBP	5.7 ± 1.20	13.7 ± 1.65	8.9 ± 1.05	0.2 ± 0.20
Number of Lob1				
Control	0	30.1 ± 4.58	43.4 ± 5.74	42.7 ± 6.41
BBP	0	39.0 ± 7.29	42.9 ± 4.43	46.2 ± 8.64

Maturation parameters and histoarchitectural study of the mammary gland for controls and rats exposed neonatally/prepubertally to BBP. Results are indicated as mean ± SEM. BW: body weight. *: significantly different compared to control group (p < 0.05).

### Mammary gland architecture

Histoarchitectural analysis of the mammary gland was performed by determining the number of specific epithelial structures at 21, 35, 50 and 100 days. The number of undifferentiated TEB decreased from 21 to 100 days in both control and BBP treated animals, while the TDs and Lob1 increased over time. Neonatal/prepubertal exposure to BBP did not modify significantly the number of these epithelial structures when compared to control groups (Table 1).

### Proliferative index of the mammary gland

**Figure 1:**
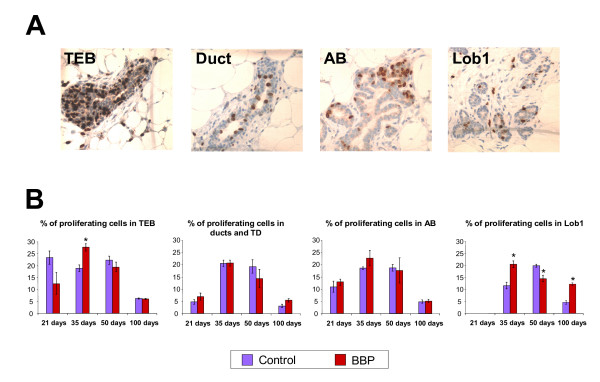
**Proliferative index analyses of the mammary gland**. A: Immunohistochemical detection of BrdU incorporation in proliferating cells (brown cells) in the epithelial structures TEB, TD and ducts, AB and Lob1. Olympus BX40 microscope with 40× objective. B: Proliferative index (mean ± SEM) in each epithelial structure at 21, 35, 50 and 100 days of age. *: significantly different compared to control group (p < 0.05).

BrdU incorporation into the DNA was used as an index of cell proliferation (Figure 1). TEB from BBP exposed rats had significantly (p < 0.05) higher proliferation index than controls by 35 days, whereas in TD and AB the differences were not significant. The proliferative index of Lob1 from controls increased from 35 to 50 days and decreased at 100 days, while in BBP exposed rats this structure showed maximum index of proliferation at 35 days and decreased thereafter, showing significant differences when compared to controls at all these ages (Figure 1).

### Gene expression analysis by microarrays

**Figure 2:**
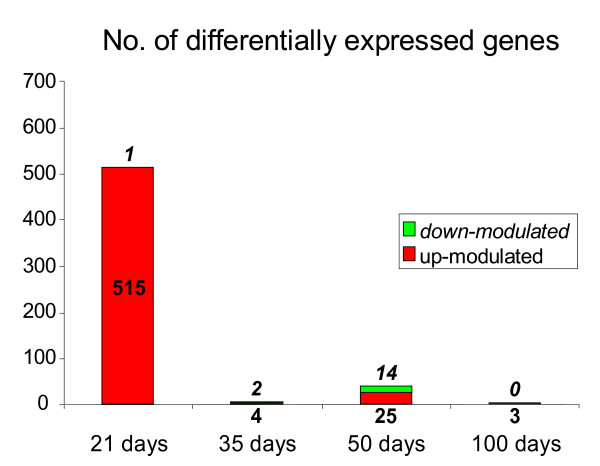
**Genomic changes induced by BBP**. Number of differentially expressed genes in the mammary glands of rats exposed neonatally/prepubertally to BBP when compared to controls at different ages. Genes were obtained by confident analysis of microarrays data at p < 0.05.

Neonatal/prepubertal exposure to BBP induced changes in the gene expression pattern of the mammary gland mainly by 21 days, just after the end of the treatment, and the number of modulated genes was low thereafter (Figure 2). In 21 days-old rats, BBP exposure resulted in the up-regulation of 515 genes, 141 of which were known, and included genes related to morphogenesis and cell differentiation, transcription factors, cell proliferation, response to stress, signal transduction, metabolism, transport and cell organization (Table 2). There was only one down-regulated gene (*gad1*). At 35 days of age the number of up-regulated genes decreased significantly to four unknown; two genes were down-regulated (one known -*gad1*-). By 50 days of age, 25 genes were up-regulated (10 known), and 14 genes were down-regulated (eight known). In the 100 day-old group, two known and one unknown genes were up-modulated (Table 2).

**Table 2:** Microarrays results

**Gene name**	**Symbol**	**Fold change BBP/Control**	**Gene ID**
**21 days**			
***Up-regulated genes***			
*Morphogenesis, organogenesis and cell differentiation*			
Cadherin 1	Cdh1	2.21	NM_031334
Cadherin EGF LAG seven-pass G-type receptor 3	Celsr3	2.16	NM_031320
Cardiotrophin 1	Ctf1	1.98	NM_017129
Early growth response 2	Egr2	2.03	NM_053633
Endothelial cell-specific molecule 1	Esm1	1.91	NM_022604
Forkhead box G1	Foxg1	2.21	NM_012560
Gamma-aminobutyric acid (GABA) B receptor 1	Gabbr1	2.14	AA817879
Glutamate receptor, metabotropic 5	Grm5	2.52	NM_017012
Glypican 2 (cerebroglycan)	Gpc2	1.83	NM_138511
NAD(P)H dehydrogenase, quinone 2	Nqo2	1.94	AW918463
Odd Oz/ten-m homolog 2 (Drosophila)	Odz2	2.15	NM_020088
Parathyroid hormone-like peptide	Pthlh	2.03	NM_012636
Pregnancy-induced growth inhibitor	Okl38	2.29	NM_138504
RAS-like family 11 member B	Rasl11b	1.96	BQ202027
Tenascin R	Tnr	1.93	NM_013045
Wilms tumor 1	Wt1	2.77	NM_031534
*Other transcription factors*			
CUG triplet repeat, RNA-binding protein 2	Cugbp2	2.00	BE112513
Forkhead box A1 (Hnf3a)	Foxa1	2.24	NM_012742
HNF-3/forkhead homolog-1 (Hfh1)	Hfh1	2.08	NM_022858
Jun dimerization protein 2	Jundp2	2.05	NM_053894
Nuclear transcription factor-Y gamma	Nfyc	2.26	NM_012866
TEA domain family member 2	Tead2	1.87	AI043589
Transcription elongation factor A (SII), 2	Tcea2	1.99	NM_057098
Transcription factor 2	Tcf2	2.04	NM_013103
Transcription factor 4	Tcf4	2.13	BF398507
*Cell proliferation*			
Alpha-2-glycoprotein 1, zinc	Azgp1	2.08	NM_012826
Calcium/calmodulin-dependent protein kinase II beta subunit	Camk2b	1.83	NM_021739
Chemokine (C-X-C motif) ligand 10	Cxcl10	1.84	NM_139089
Insulin receptor substrate 2	Irs2	2.28	CB547418
Neutrophil cytosolic factor 1	Ncf1	2.64	NM_053734
Platelet-derived growth factor, C polypeptide	Pdgfc	1.94	NM_031317
Upregulated by 1,25-dihydroxyvitamin D-3	Txnip	2.58	CB548021
*Response to stress*			
Aryl hydrocarbon receptor	Ahr	1.95	NM_013149
Chemokine (C-C motif) ligand 5	Ccl5	2.05	NM_031116
Fc receptor, IgE, low affinity II, alpha polypeptide	Fcer2a	1.94	NM_133550
Heat shock 27 kD protein family, member 7	Hspb7	2.20	CB606395
Heat shock factor 2	Hsf2	2.43	NM_031694
Myeloid cell leukemia sequence 1	Mcl1	1.84	BF553322
Nitric oxide synthase 2, inducible	Nos2	1.83	NM_012611
Oxidized low density lipoprotein (lectin-like) receptor 1	Oldlr1	2.09	NM_133306
Peptidoglycan recognition protein 1	Pglyrp1	1.99	NM_053373
Thioredoxin reductase 1	Txnrd1	2.24	NM_031614
*Signal transduction*			
5-hydroxytryptamine (serotonin) receptor 2A	Htr2a	1.96	NM_017254
Activin receptor interacting protein 1	Acvrip1	2.24	NM_053621
Agouti	A	2.18	NM_052979
Angiotensin receptor-like 1	Agtrl1	2.06	NM_031349
Growth factor receptor bound protein 2-associated protein 2	Gab2	1.92	NM_053417
Growth hormone releasing hormone	Ghrh	1.98	NM_031577
Guanylate cyclase 1, soluble, beta 2	Gucy1b2	1.91	NM_012770
MAS-related G-protein coupled receptor, member G	Mrgprg	2.80	CB545776
Mcf.2 transforming sequence-like	Mcf2l	2.24	NM_053951
Membrane-spanning 4-domains, subfamily A, member 2	Ms4a2	1.88	NM_012845
Protein kinase, cAMP-dependent, regulatory, type 2, alpha	Prkar2a	2.01	AW919085
Rho guanine nucleotide exchange factor (GEF) 1	Arhgef1	2.09	NM_021694
Rho guanine nucleotide exchange factor 7	Arhgef7	1.88	CA512056
*Cell-cell signaling*			
4-aminobutyrate aminotransferase	Abat	1.85	BF393840
Discs, large (Drosophila) homolog-associated protein 2	Dlgap2	2.38	NM_053901
Gap junction membrane channel protein beta 5	Gjb5	1.89	NM_019241
*Metabolism*			
11-beta-hydroxysteroid dehydrogenase	11-HSDIB	1.93	M77835.1
Acetyl-coenzyme A carboxylase alpha	Acaca	2.09	NM_022193
Aldehyde oxidase 1	Aox1	2.39	NM_019363
Alpha 1,2-fucosyltransferase C gene	Ftc	1.98	NM_020541
Apolipoprotein A-I	Apoa1	2.50	NM_012738
Cholinergic receptor, muscarinic 3	Chrm3	2.08	NM_012527
Diazepam binding inhibitor-like 5	Dbil5	1.84	NM_021596
Elastase 2	Ela2	2.03	NM_012553
Fatty acid binding protein 3	Fabp3	2.14	NM_024162
FK506 binding protein 1b	Fkbp1b	2.01	NM_022675
Glucosidase, alpha; acid (glycogen storage disease type II)	Gaa	2.30	CB544857
Hydroxy-delta-5-steroid dehydrogenase, 3 beta- and steroid delta-isomerase 1	Hsd3b1	1.94	AI060276
NADH dehydrogenase (ubiquinone) 1 alpha subcomplex 5	Ndufa5	2.03	BQ210630
Nicotinamide nucleotide transhydrogenase	Nnt	1.95	CB544867
Peptide/histidine transporter PHT2	Slc15a3	1.92	NM_139341
Phosphate cytidylyltransferase 1, choline, alpha isoform	Pcyt1a	2.49	NM_078622
Procollagen-lysine, 2-oxoglutarate 5-dioxygenase 1	Plod1	2.43	AA944202
Proteasome (prosome, macropain) 26S subunit, ATPase 3	Psmc3	1.90	NM_031595
Protein kinase C, gamma	Prkcc	2.04	NM_012628
Serine/threonine kinase 2	Slk	1.89	AA955942
Small glutamine rich protein with tetratricopeptide repeats 2	Sgtb	2.03	CB545680
Succinate-semialdehyde dehydrogenase		1.93	L34821.1
Transmembrane protease, serine 8 (intestinal)	Tmprss8	1.88	CB544856
Valosin-containing protein	Vcp	2.44	CB546610
*Transport*			
Adducin 1 (alpha)	Add1	2.65	NM_016990
Apolipoprotein A-V	Apoa5	2.00	NM_080576
Cholinergic receptor, nicotinic, alpha polypept. 1	Chrna1	1.88	NM_024485
CUB and zona pellucida-like domains 1	Cuzd1	2.25	NM_054005
Epidermal growth factor	Egf	2.05	NM_012842
Gamma-aminobutyric acid A receptor, gamma 2	Gabrg2	1.87	AW523484
Glutamate receptor, ionotropic, kainate 4	Grik4	2.00	NM_012572
Glutamate receptor, ionotropic, NMDA2D	Grin2d	1.91	NM_022797
Olfactory receptor 226	Olfr41	1.90	NM_031710
Potassium inwardly-rectifying channel, subfamily J, member 6	Kcnj6	1.84	NM_013192
RAB10, member RAS oncogene family	Rab10	2.33	CB547865
Rabaptin, RAB GTPase binding effector protein 2	Rabep2	2.07	NM_058213
Sodium channel, voltage-gated, type II, beta polypeptide	Scn2b	2.01	NM_012877
Solute carrier family 12, member 4	Slc12a4	2.38	BI295055
Solute carrier family 13, member 1	Slc13a1	1.89	AA818620
Synaptotagmin 8	Syt8	2.48	NM_053325
*Cell organization*			
Adaptor-related protein complex 3, mu 2 subunit	Ap3m2	2.00	NM_133305
ATP-dependent, RNA helicase	Ddx52	1.84	NM_053525
Bassoon	Bsn	1.94	NM_019146
Cadherin-8	Cdh8	2.42	AB010436.1
Integrin alpha 5	Itga5	1.94	CB546515
Myoglobin	Mb	2.13	AA964911
Synaptotagmin 1	Syt1	2.14	AW141251
Trans-golgi network protein 1	Tgoln2	1.93	NM_138840
*Other*			
Alpha-1-B glycoprotein	A1bg	1.84	NM_022258
Aortic preferentially expressed gene 1	Apeg1	2.53	CB547339
Arfaptin 1	LOC60382	2.00	NM_021763
CDC91 cell division cycle 91-like 1 (S.cerevisiae)	Cdc91l1	2.57	BQ193826
Delta-like 1 homolog (Drosophila)	Dlk1	2.30	NM_053744
Glycoprotein, synaptic 2	Gpsn2	1.87	
Guanylate cyclase activator 2A	Guca2a	2.43	NM_013118
Heparan sulfate proteoglycan, perlecan domain I	Cd44	2.20	U56859.1
Heat-responsive protein 12	Hrsp12	1.92	NM_031714
Insulin-like 6	Insl6	1.95	NM_022583
Kinesin-related protein KRP5	KRP5	1.99	AF035954.1
Luteinizing hormone beta	Lhb	1.99	U25802
Ly6-C antigen	Ly6c	2.02	NM_020103
Mitochondrial capsule selenoprotein	Mcsp	2.46	NM_031536
Neural visinin-like Ca2+-binding protein type 2	Nvjp2	1.95	NM_017357
NIMA related kinase 2	Nek2	1.86	AF352021.1
Nuclear GTPase PIKE	PIKE	1.84	NM_023026
Nucleoporin 98	Nup98	2.18	NM_031074
Oxytocin receptor	Oxtr	2.11	AF380129.1
Olfactory-specific cytochrome P-450	P-450olf1	2.03	M33296.1
Potassium channel tetramerisation domain containing 13	Kctd13	2.33	AI045333
Proline-rich protein 15	Prp15	2.24	NM_012632
RAB8A, member RAS oncogene family	Mel	2.12	BC087584
Regucalcin gene promotor region related protein	Rgpr	2.05	AB060653
Ribonuclease L (2',5'-oligoisoadenylate synthetase-dependent)	Rnasel	2.05	CB546885
Ring finger protein 38	Oip1	1.85	NM_134467
Serine protease inhibitor	Spin2b	2.67	NM_012657
Signal recognition particle 54 kDa	Srp54	1.97	BC079117
Sodium-cotransporter rkST1	rkST1	1.95	U47673.1
Synaptosomal-associated protein, 91 kDa homolog (mouse)	Snap91	2.23	NM_031728
Telomerase catalytic subunit		2.61	AF247818.1
Titin	Ttn	1.88	AF059344.1
Ubiquitin specific protease 11	Usp11	2.51	CB546595
Zinc finger protein 14 (DZF14)	DZF14	1.87	U78142.1
Zinc finger, DHHC domain containing 7	Zdhhc7	1.83	AY040615
***Down-regulated genes***			
Glutamate decarboxylase 1	Gad1	-3.65	NM_017007
**35 days**			
Glutamate decarboxylase 1	Gad1	-2.69	NM_017007
**50 days**			
***Up-regulated genes***			
Carboxylesterase 1	Ces1	1.79	NM_031565
Carboxypeptidase A1 (pancreatic)	Cpa1	1.74	NM_016998
Corticotropin releasing hormone 1	Crhr1	1.79	NM_030999
Ectonucleotide pyrophosphatase/phosphodiesterase 5	Enpp5	2.00	CB544850
Glucagon receptor, transcript variant 2	Gcgr	1.74	NM_172091
Glycoprotein 2 (zymogen granule membrane)	Gp2	1.80	NM_134418
Malic enzyme 1	Me1	1.74	NM_012600
Nuclear receptor subfamily 4, group A, member 1	Nr4a1	2.06	NM_024388
Transcription elongation factor A2	Tcea2	1.75	NM_057098
Tryptophan hydroxylase	Tph	1.93	X53501.1
***Down-regulated genes***			
Actinin alpha 3	Actn3	-2.76	NM_133424
Creatine kinase, muscle form	Ckm	-3.64	NM_012530
Glutamate decarboxylase 1	Gad1	-2.69	NM_017007
Myosin, light polypeptide 2	Myl2	-4.90	NM_012605
Parvalbumin (calcium binding protein)	Pvalb	-2.38	NM_022499
Titin	Ttn	-2.51	AF059344.1
Troponin I, skeletal, fast 2	Tnni2	-2.46	NM_017185
Troponin-c	Tnnc2	-4.77	AI639532

**100 days**			

Translin	Tsn	2.40	NM_021762
Whey acidic protein	Wap	2.87	NM_053751

List of known genes down- and up-regulated in mammary glands of BBP treated rats at 21, 35, 50 and 100 days. Average fold change related to control mammary glands is indicated. Up-regulated genes at 21 days have been sorted by function according to level 4 of Gene Ontology.

Functional analysis of the genes found up-regulated in 21 days-old rats showed some categories that were significantly (p < 0.05) over-represented: cell proliferation and differentiation, signal transduction (TGF-beta signaling pathway, metabotropic glutamate receptor group I pathway, PI3 kinase pathway, endothelin signaling pathway and interleukin signaling pathway) and cell communication, (neuronal activities, anterior/posterior patterning). We also found over-representation of genes coding for cytoskeletal proteins among the down-modulated by 50 days.

### Validation of microarrays by real time RT-PCR

**Figure 3:**
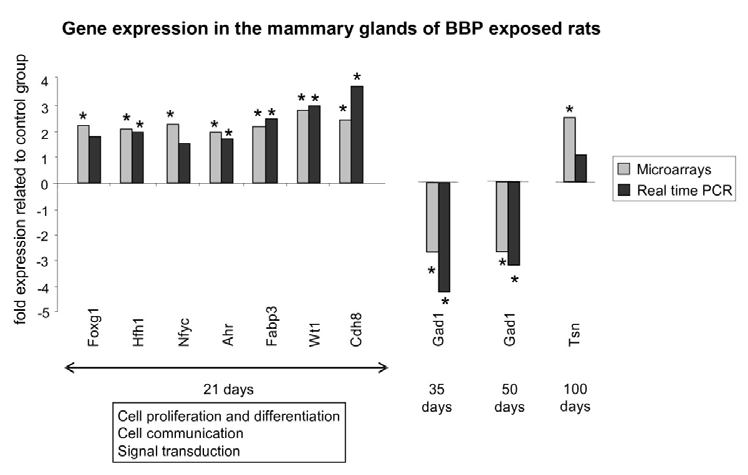
**Comparative expression analyses of selected genes**. Gene expression analyses by real time RT-PCR in comparison with results from microarrays. Grey bars represent results obtained in the microarrays, black bars represent results obtained in the real time RT-PCR. Results show the mean fold expression of the treated group related to control group. The functional categories significantly over-represented are indicated (21 days). *: significant differences compared to control group (p < 0.05).

To validate the results from microarrays we have chosen several genes that were differentially expressed in the mammary glands from BBP treated rats: transcription factors related to proliferation and differentiation (Foxg1, Hfh1, Nfyc, Ahr, Wt1, Tsn), other genes related to differentiation (Fabp3), adhesion (Cdh8) and communication (Gad1). We analyzed the gene expression of those genes by real time RT-PCR. Results of the validation are shown in Figure 3. In the BBP exposed animals Foxg1, Hfh1, Nfyc and Ahr were between 1.6- and 2-fold up-regulated in comparison to their gene expression in untreated animals. These differences, although discrete, were statistically significant for Hfh1 and Ahr, and close to significance for the other genes. We also obtained statistically significant increases in the expression of Fabp3, Wt1 and Cadh8. By 35 and 50 days, BBP exposure induced a significant decrease in the expression of Gad1. The decrease of this gene found by microarrays at 21 days as well as the increase in Tsn observed by 100 days of age was not confirmed by real time PCR.

## Discussion

The objective of the present study was to investigate the effects of neonatal/prepubertal exposure to BBP in the rat mammary gland at different times of development. We have characterized the genomic signature of the mammary gland, observing modifications in the gene expression profile mainly at the end of the exposure and fewer changes thereafter. We also studied maturation parameters, finding also alterations compatible with a transitory effect of this environmental compound.

Although it is difficult to determine doses for experimental studies, the dose chosen for this study was based on the work of Singletary et al. [17], where they reported that treatment of female rats with BBP for 7 days prior to dosing with DMBA decreased mammary tumor incidence by 37%. The number of palpable tumors per rat at week 15 was significantly inhibited by 58% and 71% for animals administered BBP at 250 and 500 mg/kg, respectively, compared to controls. Our goal was to determine if neonatal and prepubertal only exposure to BBP would have a direct effect at day 21 and if this treatment would leave permanent alterations on mammary gland maturation, cell proliferation and gene expression at later time points. The offspring were exposed to the BBP via the mother's milk. Considering that the lactating dams were treated with 500 mg BBP/kg BW and this is dissipated in the mother before it makes its way via the milk to the 10 offspring, hence it is estimated that they receive 1/100 to 1/1000 of the mother's dose, i.e. 0,5–5 mg BBP/kg BW/day. This approximation places the rat BBP exposure near the EPA safe dose for humans of 0.2 mg/kg/day [18] and similar to a 2 μg/kg BW/day for adults, and lower than estimated exposure to infants and children (6 μg/kg BW/day) [1].

Neonatal/prepubertal exposure to BBP increased the relative uterine weight at 21 days but not thereafter, and decreased the body weight at day of vaginal opening (approximately day 30). In the literature there are conflicting results regarding the effect of BBP because it showed estrogenic effects *in vitro *but did not modify the estrogen-modulated gene calbindin-D9k *in vivo *[19]. However, BBP has also shown to increase the expression of progesterone receptor mRNA, a gene regulated by estrogens, in the preoptic area of adult ovariectomized rats [20]. Exposure to BBP in other phases of development induced alterations in the onset of puberty. Ashby *et al*. reported advanced vaginal opening by 1.1 days without modifications on uterine weight in rats by effect of prenatal plus prepubertal exposure to BBP, although the authors considered that this result could be related to the variability of the observation or, more interestingly, to an increase in the weight of the BBP treated animals [21]. In two generational studies, Aso *et al*. reported changes in anogenital distance of female offspring of rats treated with more than 100 mg/kg [22].

Little is known on the effects of BBP on mammary gland, another target of sexual maturity, and the present work indicates that BBP did not induce significant changes in the morphology of that gland, but changed the proliferative index of TEB by 35 days and in Lob1 at 35, 50 and 100 days. Although these modifications are subtle, we cannot rule out that they may have an effect if the mammary gland susceptibility to carcinogenesis, since TEB are the most susceptible epithelial structures to malignant transformation [23] and prevention of chemically-induced mammary carcinomas is accompanied by inhibition of cell proliferation [13,14]. Changes in the proliferative index of Lob1 in BBP exposed animals in comparison to control group can be associated with subtle alterations in mammary development. The proliferation of Lob1 is related to the differentiation of these structures to lobules type 2 [23]. Control group had the highest proliferative index in Lob1 by 50 days, concomitantly with the highest number of Lob1, at the age of profound changes that the mammary gland faces as a result of an intense hormonal activity [13,14]. BBP exposed animals had the maximum mitotic index in Lob1 by 35 days, decreasing by 50 days. In addition, the reduction of proliferation in Lob1 of BBP group by 100 days was not as important as in control group. The significant changes observed are compatible with slight changes in the timing of the breast tissue development by effect of BBP exposure.

We are the first to report that neonatal/prepubertal exposure to BBP induced modifications in the gene expression of the mammary tissue. Whereas the full relevance of these findings requires tumorigenesis studies, the characterization of the genomic signature of the mammary gland could be used as a predictor of the susceptibility to carcinogenesis. This statement is based on previous works in which changes in susceptibility to DMBA-induced carcinogenesis are accompanied by the induction of a specific genomic profile [13,14,24]. Neonatal/prepubertal exposure to BBP induced changes in the genomic profile of the mammary gland, mainly at the end of treatment (21 days) with a focus on genes involved in cell proliferation/differentiation, communication and signal transduction. Some of those genes related to differentiation were validated by real time RT-PCR, as Fabp3 and Wt1. Fabp3 belongs to the family of the fatty acid binding proteins, which have a role in fatty acid transport or metabolism. This member, however, is considered an identical homologue of the mammary-derived growth inhibitor (MDGI), with a role in the differentiation of the mammary gland [25]. MDGI is a suppressor of tumor growth and an inhibitor of tumor cell proliferation [26]. We have previously observed that this gene is up-regulated in the most differentiated structures of the mammary gland (lobules type 4) and silenced in breast cancer progression [27]. Another gene found significantly up-regulated by 21 days is Wt1, which has also been related to development of the mammary gland although its specific function is not well known [28,29]. This gene is essential for the development of the urogenital system but its role in the breast tissue is controversial. Wt1 presents a developmental pattern of expression in normal mammary gland, and changes observed in cell lines and tumors may be indicative of breast-tumor-related perturbations of Wt1 expression [29]. Cadherin 8 (cdh8), also found up-regulated in the exposed animals, is an adhesion molecule with an important role in brain and kidney development and differentiation [30,31]. We have also observed transitional changes in the expression of these three genes by effect of prepubertal exposure to other endocrine disruptor, bisphenol A [32]. Moreover, we also observed changes in the expression of Fabp3 when this endocrine disruptor was administered *in utero*. Thus, the exposure during the prenatal period to bisphenol A induced up-regulation or down-regulation of Fabp3 depending on the age tested, suggesting a "shift" in the normal development of the mammary gland, while prepubertal exposure to this endocrine disruptor induced a transient up-regulation just at the end of the treatment [32]. Fabp3 mRNA has also been reported as sensitive to the action of other hormone interfering agents, since the antiestrogens tamoxifen and ICI up-regulate its expression by stabilization of the mRNA [33]. These evidences suggest that genes related to differentiation of the mammary gland can be very sensitive to the effects of several compounds that may interfere with hormonal action, suggesting that, even if the effects are not strong enough to modify mammary histoarchitecture, they may change the molecular milieu of the gland.

By 21 days of age we also found a moderate increase in the expression of the transcription factors Foxg1, Hfh1 and Ahr. Foxg1 and Hfh1 have a role in the development and differentiation of several tissues, but to date their function in the mammary gland is unknown [34,35]. The aryl hydrocarbon receptor (Ahr) belongs to the PAS (per-arnt-sim) family of transcription factors. PAS proteins control important physiological processes, as toxicant metabolism, circadian rhythms and hormone signaling [36]. We have previously reported a role of Ahr in mammary differentiation, as an increased expression in primary cultures of Lob3 from parous women compared to primary cultures of Lob1 from nulliparous women breast was observed [37]. Ahr is known to interact with signaling pathways that are mediated by estrogen receptor and other hormone receptors. The mechanisms of Ahr-estrogen receptor crosstalk seem to be complex, and recent studies suggest direct activation of estrogen-regulated target genes by Ahr [38]. Thus, our results suggest that the increase in Ahr could be one of the mechanisms by which BBP treatment exerts the observed transitory effects at 21 days.

We also observed and validated down-regulation of Gad1 by 35 and 50 days of age. This gene codes for the protein Gad67, which catalyzes the production of the neurotransmitter GABA. Gad1 can be regulated by estrogens in rat brain [39,40], and expression changes in discrete regions of the rostral preoptic area during estrous cycle and with age has been described [41]. In addition, Gad1 has also a role in the control of puberty in nonhuman primates [42]. Modifications in the expression of Gad1 has been investigated in relation to some brain disorders, since Gad1 mRNA decreased in prefrontal cortex and other brain areas of schizophrenia and bipolar disorder patients with psychosis [43]. Experiments in rats have found down-modulation of Gad1 accompanied by hypermethylation of Gad1 promoter very likely mediated by the overexpression of DNA methyltransferase 1 in cortical GABAergic interneurons [44]. Thus, we can consider the possibility that the observed changes in Gad1 are due to an effect of BBP on the methylation of Gad1 promoter. BBP has also shown an effect on the methylation status of other genes *in vitro*, as the reported demethylation of ERα promoter-associated CpG islands in MCF-7 cells [45]. The GABA-ergic system has been involved in hormonal regulation and pathogenesis of breast cancer, since human and mouse tumors have shown higher GABA level and GAD activity than normal tissue [46]. Hence, although prepubertal exposure to BBP seems to have mainly a transitory effect on the genomic profile of the mammary gland, we cannot discard that subtle changes in the gland may have an effect later in life. Finally, very few genes were regulated by BBP exposure in the oldest age tested (100 days).

## Conclusion

In summary, neonatal/prepubertal exposure to BBP had a transitory effect by increasing the relative uterine weight only at the end of exposure. This compound also changed the gene expression profile of the mammary gland mainly at the same age, although long-term subtle alterations can not be ruled out.

## Methods

### Animals and experimental design

Animals were housed in 12 hour light/dark cycle and controlled temperature in the University of Alabama at Birmingham animal facility. Pregnant Sprague Dawley CD rats (Charles River, Raleigh NC) were bred and maintained on phytoestrogen-free AIN-93G diet (Harlan Teklad, Madison WI). After parturition, litters were adjusted to 10 offspring per mother, and the lactating dams (10 dams per group) were gavaged with 500 mg BBP/kg body weight or an equivalent volume of sesame oil on days 2–20 (Mondays-Fridays only). We used a stock solution of 200 mg BBP/ml sesame oil, and dams were gastric intubated with 2,5 μl BBP/g BW (0.5 ml for a dam of 200 g) each day, preferable each late afternoon. The offspring were weaned at day 21 and continued on AIN-93G diet until day 70 where they were switched to AIN-93M diet. Vaginal opening was assessed as an index of maturation and body weight was recorded from a minimum of 27 female offsprings/group.

Female offspring were processed at 21, 35 ± 1, 50 ± 1 and 100 ± 2 days. For the cycling animals (the latter 3 ages), all females were killed in the estrus phase of the estrous cycle. The day of euthanasia the animals were anesthetized with ketamine (10 mg/100 g)/xylazine (1.5 mg/100 g), the fourth mammary glands were rapidly dissected and frozen for microarrays analysis (1 female offspring from each litter at each time point). Transsection of the aorta was performed after the tissues were properly collected. From another set of females from each litter, mammary glands were dissected for whole mount preparation and gland differentiation analysis, and the contralateral mammary glands were paraffin-blocked for cell proliferations studies. Ten glands representing all litters per group at each time point were used for each of the molecular, morphological and proliferation analyses. Uterine weight was also recorded at day of sacrifice and uterine weight/body weight ratios were calculated for each animal. Differences among groups were analyzed using Tukey's Test (p < 0.05). All animal studies were conducted in accordance with the University of Alabama at Birmingham Guidelines for Animal Use and Care.

### Mammary whole mounts analysis

The excised mammary glands were fixed in 10% neutral-buffered formalin followed by de-fatting in acetone. The tissues were stained in alum carmine overnight and dehydrated in a series of graded ethanol. The glands were cleared in xylene and coverslipped with mounting media. Analysis of mammary epithelial structures (terminal end buds -TEBs-, terminal ducts -TDs-, alveolar buds -ABs- and lobules type 1 -Lob1-) was made through visual evaluation and computer-assisted image analysis using MetaMorph Imaging System (Universal Imaging Corporation, Downingtown PA). The outer 5-mm margin of the mammary whole mount was examined by light microscopy using the criteria of Russo and Russo [23]. This area represents the location of most actively proliferating TEB structures of the mammary gland for a young virgin rat [47], The total number of each epithelial structure was determined under an Olympus microscope using a 40× magnification objective. The collected data were analyzed using two-tailed unpaired t-tests.

### Proliferative index analysis

Two hours before euthanasia animals were weighed and injected i.p. with 100 mg bromodeoxyuridine (BrdU, Sigma, St. Louis, MO) per kg body weight. The fourth abdominal mammary glands were excised and fixed in 10% neutral-buffered formalin, embedded in paraffin and sectioned at 4 μm thickness. Tissue sections were mounted on positively charged slides and immunocytochemically reacted with anti-BrdU monoclonal antibody (BioGenex, San Ramon, CA) using an automatic slide stainer (BioGenex). The incorporation of BrdU in the mammary glands was visualized using the streptavidin-biotin labeling system with 3, 3'-diaminobenzidine (DAB) as a color reaction substrate (BioGenex). The proliferative index was determined quantitatively using an Olympus BX40 microscope (60× magnification objective) as the percentage of DAB positive cells within each specific epithelial structure, i.e., TEBs, TD and ducts, ABs and Lob1. All cells in each structure were counted blinded to the groups. A minimum of 1,000 cells per tissue sample was examined. Data from different groups were analyzed by unpaired t-tests.

### Characterization of the genomic profile

Gene expression profile was determined from total RNA using Agilent 60-mer oligo-microarrays slides containing 22,000 sequences. RNAs from the mammary glands were extracted with the RNeasy Mini Kit (Qiagen Inc., Valencia, CA). The quantity and purity of each RNA sample was determined from the 260- and 280-nm absorbance, and its integrity was determined (2100 BioAnalyzer; Agilent Technologies Inc., Palo Alto, CA). Only high quality RNA, with absorbance 260/280 greater than 1.8 and RNA integrity number (RIN) greater than 8 were considered for further analysis. 250 ng of pooled RNA from two-three different mammary glands were fluorescent labeled with Agilent Low Input RNA Fluorescent Linear Amplification Kit (Agilent Technologies) in the presence of Cy3-dCTP or Cy5-dCTP (Perkin Elmer, Wellesley, MA), and purified using RNeasy Mini Kit (Qiagen Inc., Valencia, CA).

Four separate experiments, each including two to three mammary gland cRNA pool were performed at each time point studied. 750 ng of Cy3 labeled control cRNA and 750 ng of Cy5 labeled BBP group cRNA were used to hybridize each slide at 60°C for 18 hours. Slides were washed and fluorescence images were captured with a laser scanner (Agilent Technologies). The obtained images were analyzed with Feature extraction software (Agilent Technologies) to verify the quality of the hybridization by the report of the outlier data.

Feature extraction software automatically flagged non-uniform features and population outliers, considering acceptable outlier numbers lower than 5% of total feature number on the array. Intensity of each fluorescent signal was quantified by ImaGene 5.6 software (BioDiscovery Inc., El Segundo, CA) and statistically analyzed using GeneSight 4.1.6 software (BioDiscovery) with Lowess normalization considering the arrays per group as replicates. We determined the genes with ≥ 1.4-fold differences by confidence analyses at p < 0.05 following manufacturer's recommendations. We performed functional analysis of the genes obtained as modulated by BBP exposure using PANTHER (Protein ANalysis THrough Evolutionary Relationships). Genes were annotated according to known biological process, molecular function and biological pathways. The number of genes in each category was statistically compared to NCBI: *R. norvegicus *genes database to look for under- and over-represented functional categories using the binomial test [48] and the Bonferroni correction for multiple testing. Genes with known function were also classified using Gene Ontology database.

### Gene expression analysis by real-time RT-PCR

Several genes found differentially expressed in the mammary glands of rats exposed to BBP were chosen for confirmation by real-time reverse transcription PCR (RT-PCR). Beta-actin was used as a control. All RT-PCR reactions were performed on the ABI Prism 7700 Sequence Detection System (Applied Biosystems, Foster City, CA) using the fluorescent TaqMan methodology (TaqMan One Step RT-PCR Master Mix Reagents, Applied Biosytems) following manufacturer's protocol. 100 ng of total RNA in 50 μl of final volume was reverse-transcribed (30 min at 48°C) and amplified following 1 cycle of 10 min at 95°C, and 40 cycles of 15 s denaturation at 95°C and 60 s annealing at 60°C. Each gene was normalized to beta-actin and data was analyzed using two-tailed unpaired t-test.

## Abbreviations

AB: alveolar bud; 

BBP: butyl benzyl phthalate; 

Lob1: lobule type 1; 

PCR: polymerase chain reaction;

RT: reverse transcription; 

TD: terminal duct; 

TEB: terminal end bud.

## Authors' contributions

RM performed the molecular experiments and contributed in the writing of the manuscript. RW carried out the morphological and proliferative index analyses. IHR participated in the design and coordination of the study, and helped in the writing of the manuscript. DAM and GAB participated in the analysis of the microarrays. CAL participated in the coordination of the study and performed the animal assays. JR conceived of the study, coordinated the work and helped in the writing of the manuscript. All authors have read and approved the final manuscript.
